# A comparative genome analysis of Rift Valley Fever virus isolates from foci of the disease outbreak in South Africa in 2008-2010

**DOI:** 10.1371/journal.pntd.0006576

**Published:** 2019-03-21

**Authors:** Moabi R. Maluleke, Maanda Phosiwa, Antoinette van Schalkwyk, George Michuki, Baratang A. Lubisi, Phemelo S. Kegakilwe, Steve J. Kemp, Phelix A. O. Majiwa

**Affiliations:** 1 ARC-Onderstepoort Veterinary Research, Gauteng, South Africa; 2 Department of Veterinary Tropical Diseases, University of Pretoria, Gauteng, South Africa; 3 International Livestock Research Institute, Nairobi, Kenya; 4 Department of Agriculture, Land Reform and Rural Development, Veterinary Services, Northern Cape Province, South Africa; George Mason University, UNITED STATES

## Abstract

Rift Valley fever (RVF) is a re-emerging zoonotic disease responsible for major losses in livestock production, with negative impact on the livelihoods of both commercial and resource-poor farmers in sub-Sahara African countries. The disease remains a threat in countries where its mosquito vector thrives. Outbreaks of RVF usually follow weather conditions which favour increase in mosquito populations. Such outbreaks are usually cyclical, occurring every 10–15 years. Recent outbreaks of the disease in South Africa have occurred unpredictably and with increased frequency. In 2008, outbreaks were reported in Mpumalanga, Limpopo and Gauteng provinces, followed by 2009 outbreaks in KwaZulu-Natal, Mpumalanga and Northern Cape provinces and in 2010 in the Eastern Cape, Northern Cape, Western Cape, North West, Free State and Mpumalanga provinces. By August 2010, 232 confirmed infections had been reported in humans, with 26 confirmed deaths.To investigate the evolutionary dynamics of RVF viruses (RVFVs) circulating in South Africa, we undertook complete genome sequence analysis of isolates from animals at discrete foci of the 2008–2010 outbreaks. The genome sequences of these viruses were compared with those of the viruses from earlier outbreaks in South Africa and in other countries. The data indicate that one 2009 and all the 2008 isolates from South Africa and Madagascar (M49/08) cluster in Lineage C or Kenya-1. The remaining of the 2009 and 2010 isolates cluster within Lineage H, except isolate M259_RSA_09, which is a probable segment M reassortant. This information will be useful to agencies involved in the control and management of Rift Valley fever in South Africa and the neighbouring countries.

## Author summary

A single RVF virus serotype exists, yet differences in virulence and pathogenicity of the virus have been observed. This necessitates the need for detailed genetic characterization of various isolates of the virus. Some of the RVF virus isolates that caused the 2008–2010 disease outbreaks in South Africa were most probably reassortants resulting from exchange of portions of the genome, particularly those of segment M. Although clear association between RVFV genotype and phenotype has not been established, various amino acid substitutions have been implicated in the phenotype. Viruses with amino acid substitutions from glycine to glutamic acid at position 277 of segment M have been shown to be more virulent in mice in comparison to viruses with glycine at the same position. Phylogenetic analysis carried out in this study indicated that the viruses responsible for the 2008–2010 RVF outbreaks in South Africa were not introduced from outside the country, but mutated over time and caused the outbreaks when environmental conditions became favourable.

## Introduction

Rift Valley fever (RVF), a mosquito-borne viral disease, affects humans and some species of ruminants including sheep, cattle, goats and buffalos. The causative agent, Rift Valley Fever virus (RVFV), belongs to the genus *Phlebovirus* in the family *Phenuiviridae* [1]. The disease in livestock is characterized by abortion storms and high mortality of young animals [2]. In humans, it manifests as febrile illness, resulting in retinal degeneration, severe encephalitis, haemorrhage; fatal hepatitis occurs in less than 1% of patients [3]. Protection of animals from the disease can be conferred by vaccination; however, there is currently no approved vaccine for use in humans. Transmission of RVF can be prevented by eliminating the mosquito vectors and avoiding human contact with tissues of infected animals.

The RVF virus has been isolated from more than 30 species of mosquitoes, belonging to at least six genera (*Aedes*, *Culex*, *Anopheles*, *Eretmapodites*, *Mansonia* and *Coquillettidia*). In a number of mosquito species, the virus has been isolated from both the insects and their eggs, suggesting different modes of transmission [4, 5]. Furthermore, the virus has been isolated from unfed mosquitoes reared from eggs obtained during inter-epidemic periods in Kenya and South Africa [4,5,6]. Eggs from floodwater mosquitoes can remain viable for considerable time between outbreaks, hatching during conducive climatic conditions associated with periods of high rainfall [4]. Changing climate and farming systems can create conditions favorable for mosquito breeding, resulting in unexpected outbreaks of the disease [7].

The disease is endemic in eastern and southern Africa, but outbreaks have been reported in Egypt, Madagascar, Mauritania, Saudi Arabia, Sudan and Yemen [8,9,10]. No significant antigenic differences have thus far been demonstrated among isolates of this virus from different geographic locations, confirming the existence of a single RVFV serotype. Despite the single serotype, differences in virulence and pathogenicity of the virus have been observed, necessitating the need for detailed genetic characterization of the various isolates [11,12].

The genome of RVFV, like that of the other *bunyaviruses*, consists of three-segmented, single-stranded negative- and ambi-sense RNAs with a total size of 12kb. The L (Large) segment codes for the viral RNA polymerase. The M (Medium) segment encodes a single precursor protein which is cleaved to produce the envelope glycoproteins G1 and G2, and two non-structural proteins of 78kDa and 14 kDa. In contrast, the ambisense S (Small) segment codes for the nonstructural protein NSs in the genomic sense and the nucleocapsid protein N in the antigenomic sense [13,14,15].

Because of its segmented structure, the genome of RVFV is thought to undergo recombination through reassortment, thereby contributing to its evolutionary dynamics [12,16]. In general, the RVFV genome is characterized by low genetic diversity (~5%); consequently, it is difficult to statistically detect intragenic recombination events [11,15]. Similar to other arboviruses, all the genes of RVFV are under purifying selection and have evolved at distinct rates by accumulating mutations at 1.9 x 10^−4^ to 2.5 x 10^−4^ substitutions per site per year [12,16]. The previously estimated time to most recent common ancestor (TMRCA) is at around 124 to 133 years. This coincides with the importation of highly susceptible European breeds of cattle and sheep into East Africa where the disease was first reported [16, 17, 18, 19].

Despite the high percentage sequence identity, nucleotide sequences of complete or partial segments from viruses isolated from various countries over the last 60 years, have been grouped into 15 lineages [20]. Based on the topologies of phylogenetic trees constructed from nucleotide sequences representing each of the three genome segments, it is suggested that reassortment, specifically of a 2010 isolate from a patient in South Africa, contributed to antigenic shift during outbreaks. The individual was accidentally co-infected with live RVF animal vaccine and a RVF virus in lineage H [20].

In 2009, a RVF outbreak with unusual clinical presentation in animals was observed in South Africa. This outbreak had two distinguishing features: first, it occurred atypically in the absence of abnormally high rainfall; secondly, in addition to causing abortion storms, it had a high mortality among pregnant adult cattle [21].

The first case of RVF in South Africa occurred in the summer of 1950–1951 in animals and subsequently it was diagnosed in humans in 1951 [22,23]. Three major outbreaks of the disease occurred in South Africa in 1950–1951, 1974–1976 and, most recently, in 2008–2011. There were minor incidents in the inter-episodic periods interspersing these outbreaks [24].

Confirmation of suspected cases of RVF in animals in South Africa is normally done at Agricultural Research Council–Onderstepoort Veterinary Research (ARC-OVR). Over time, the institute has accumulated a large collection of RVFV isolates from a majority of reported cases of the disease in South Africa. In order to obtain comprehensive information on the genetic composition of the RVF viruses (RVFVs) circulating in South Africa, we performed full genome sequence analysis of some of the viruses isolated from animals at discrete foci of the outbreaks which occurred during the 2008–2010 period. The genome sequences of these viruses were compared with those of other RVFVs from earlier outbreaks in South Africa and other countries where the disease has occurred. Furthermore, the genome sequence data generated add to the repertoire of the RVFV sequences available in the public domain databases. Such data are necessary for studies required to find new or improved technologies for management of this zoonotic disease. Overall, data presented here add to the understanding of epidemiology and ecology of RVF. The information will be useful to agencies involved in the control and management of Rift Valley fever in South Africa and the neighbouring countries.

## Methods

### Samples for genome sequencing

Organ biopsies or blood specimens from buffalo, cattle or sheep in 7 South African provinces were collected from sick or dead animals during the 2008 to 2010 RVF outbreaks. The specimens were brought to the Agricultural Research Council–Onderstepoort Veterinary Research (ARC-OVR), to be subjected to confirmatory laboratory tests for RVF. The specimens were stored at 4°C for less than 24 hours before being processed for the tests, which include virus isolation.

An outbreak operationally refers to a case of RVF confirmed in a laboratory by isolation of RVF virus, detection of RVF viral RNA or IgM antibody to RVF viral protein, in a tissue specimen from an animal found at a specific location during a season [24]. Multiple outbreaks in a season constitute an epidemic.

### Isolation, propagation and purification of RVF viruses

Vero cells in T25 tissue culture flasks (approximately 2.0×10^6^ cells/ml) were inoculated with 1/10 clarified suspensions of blood or homogenized tissue in DMEM supplemented with 100 IU/ml Penicillin, 100μg/ml Streptomycin and 0.25μg/ml Amphotericin B (Lonza, BioWhittaker), and 2% foetal bovine serum (Gibco, LifeTechnologies). The cells were incubated at 37°C with 5% CO_2_ in a humid chamber for 1 hour. Thereafter the inoculum was discarded, the cell monolayers washed twice with media, 5ml of the same medium replenished and the flasks returned in the incubator, where they were monitored daily for development of cytopathic effect (CPE). Incubation lasted 4–6 days, and up to three passages were made per sample [25]. Presence of RVFV nucleic acids in the isolates was confirmed by real-time PCR using a slight modification of an established method [26]. The isolates were aliquoted in 500μl– 1ml quantities and stored at -80°C until further use.

Optimum conditions for efficient infection of Baby Hamster Kidney (BHK 21) cells (obtained from AATC) with RVF virus were established empirically using isolate M35/74, the challenge strain of RVFV [27]. The BHK 21 cells were grown in DMEM-F12 supplemented with 5%FBS (LONZA) and 1% pen/strep Amphotericin B (LONZA). These conditions were applied to infect BHK cells at a MOI resulting in the highest viral load. The infected cells were pelleted by centrifugation at 2 500 rpm for 5 minutes and the supernatant recovered. The supernatant was committed to sequence independent single primer amplification (SISPA) [28]. Briefly, the viral particles in the supernatant were treated with 100U DNase I and 4g RNase at 37°C for 2h to remove possible host nucleic acids contamination. Viral RNA was extracted using TRIZOL LS kit (Invitrogen) according to the procedure provided by the supplier (Invitrogen). The RNA was recovered and used as the template in the first strand cDNA synthesis primed with FR26RV-N (5’GCC GGA GCT CTG CAG ATA TCN NNN NN3’ [28]. The single-stranded cDNA was the template for double-stranded cDNA synthesis using random 20mer primers and Klenow fragment of *E*. *coli* DNA polymerase. These products were subjected to PCR amplification using the 20-mer region of the above primer (FR26RV: 5’GCC GGA GCT CTG CAG ATA TC3’) in a reaction incubated in a thermocycler programmed to denature at 94°C, 2 min then 35 cycles of 94°C, 30 sec; 55°C, 30 sec; 68°C, 30s; with a final extension at 68°C, for 10 min. The SISPA products were resolved by electrophoresis in 1% agarose gels.

### *Construction* of cDNA libraries

The SISPA products ranging in size from 0.2kb to 1.5kb were recovered from agarose gels and used in the preparation of library for sequencing reactions on the Next Generation Sequencing (NGS) platforms exactly as described by the manufacturer (Roche Applied Science). The sequencing was done on the Genome Sequencer 454 platform (GSFLX; 454 Life Sciences, Roche Applied Science; http://www.454.com).

### Bioinformatics analyses of sequence data

The sequence data obtained was processed and assembled into contigs using the appropriate software set to default values (Roche/454 Newbler for 454 Life Sciences Corporation, Software Release: 2.8–20120726_1306 or CLC Genomics Workbench, QIAGEN Bioinformatics).

The data was subjected to further analyses using a combination of bioinformatics software. The nucleotide sequences were aligned using Clustal W [29] within the Molecular Evolutionary Genetics Analysis (MEGA version 6) [30] set to optimum parameters for each sequence type. The best fitting nucleotide substitution model was determined for each genome segment using MEGA 6 and then applied in all the subsequent analyses. The aligned nucleotide sequences were used in calculating the mean pairwise distances and to derive phylogenetic trees using Maximum likelihood under 1000 bootstrap iterations [31].

Evidence for possible intragenic recombination events among the isolates was sought using different methods available from RDP3 [32]. Rates of molecular evolution for individual genome segments were estimated using Bayesian Markov Chain Monte Carlo (MCMC) implemented in the BEAUTI v1.8.1, BEAST v1.8.1, Tracer and FigTree packages [33]. The substitution rates were estimated using both strict and relaxed uncorrelated lognormal molecular clock under General Time Reversible (GTR) model with gamma distribution (T4). The general Bayesian skyline coalescent prior was used and the MCMC allowed to run for sufficient number of generations (> 10 million) with sampling every 1000 states, to ensure convergence of all parameters [33].

### Rift Valley fever virus genome sequence accession numbers

The nucleotide sequences of all the segments of the RVF isolates analyzed in the current study have been deposited in GenBank with accession numbers indicated in Table 1.

**Table 1:** List of RVF virus isolates analyzed in the study, with GenBank accession numbers assigned to the nucleotide sequences of their respective L, M and S segments. With the exception of Madagascar, origin imply the South African Province and nearest town from which the isolate originated.

Isolate #/year	GenBank Accession number			Host	Tissue source	Origin
	L segment	M segment	S segment			
M48/08	KX944866	KX944843	KX944820	Bovine	Liver	Madagascar
M47/08	KX944865	KX944842	KX944819	Bovine	Liver	Warmbad, (LP)
M39/08	KX944864	KX944841	KX944818	Buffalo calf	Whole foetus	Nelspruit (MP)
M37/08	KX944863	KX944840	KX944817	Buffalo calf	Whole foetus	Hoedspruit (LP)
M85/08	KX944871	KX944848	KX944825	Bovine Calf	Carcase	Irene (GP)
M84/08	KX944870	KX944847	KX944824	Bovine	Blood	Irene (GP)
M80/08/2	KX944869	KX944846	KX944823	Bovine	Blood	Irene (GP)
M66/09	KX944868	KX944845	KX944822	bovine	Liver	Modderfontein (GP)
M260/09	KX944860	KX944837	KX944814	Bovine	Foetus organ	Upington (NC)
M259/09	KX944859	KX944836	KX944812	Bovine	Blood	Upington (NC)
M247/09	KX944857	KX944834	KX944811	Ovine	Liver, lung	Upington (NC)
M127/09	KX944851	KX944828	KX944804	Bovine	Blood, fresh liver	Cascade (KZN)
M33/10	KX944862	KX944839	KX944816	Ovine	Liver	Middleburg (EC)
M29/10	KX944861	KX944838	KX944815	Bovine	Blood	Pretoria (GP)
M25/10	KX944858	KX944835	KX944813	Ovine	Organs	Bultfontein (FS)
M12/10	KX944850	KX944827	KX944805	Ovine	Blood	Bultfontein (FS)
M19/10	KX944853	KX944830	KX944809	Ovine	Liver, Lung, Brain and spleen	Heldefontein (FS)
M16/10	KX944852	KX944829	KX944806	Ovine	Organs	Bultfontein (FS)
M06/10	KX944849	KX944826	KX944803	Bovine	Blood	Sterkfontein (EC)
M21/10	KX944856	KX944833	KX944810	Ovine	Organ pool	Bloemfontein (FS)
M57/74	KX944867	KX944844	KX944821	N/A	N/A	N/A
M1975Bov	KX944855	KX944832	KX944808	Bovine	N/A	N/A
M1955	KX944854	KX944831	KX944807	N/A	N/A	N/A

N/A: Not available (the information is unknown). LP: Limpopo Province; MP: Mpumalanga Province; GP: Gauteng Province; NC: Northern Cape Province; KZN: KwaZulu Natal Province; EC: Eastern Cape Province; FS: Free State Province.

## Results

**Fig 1:**
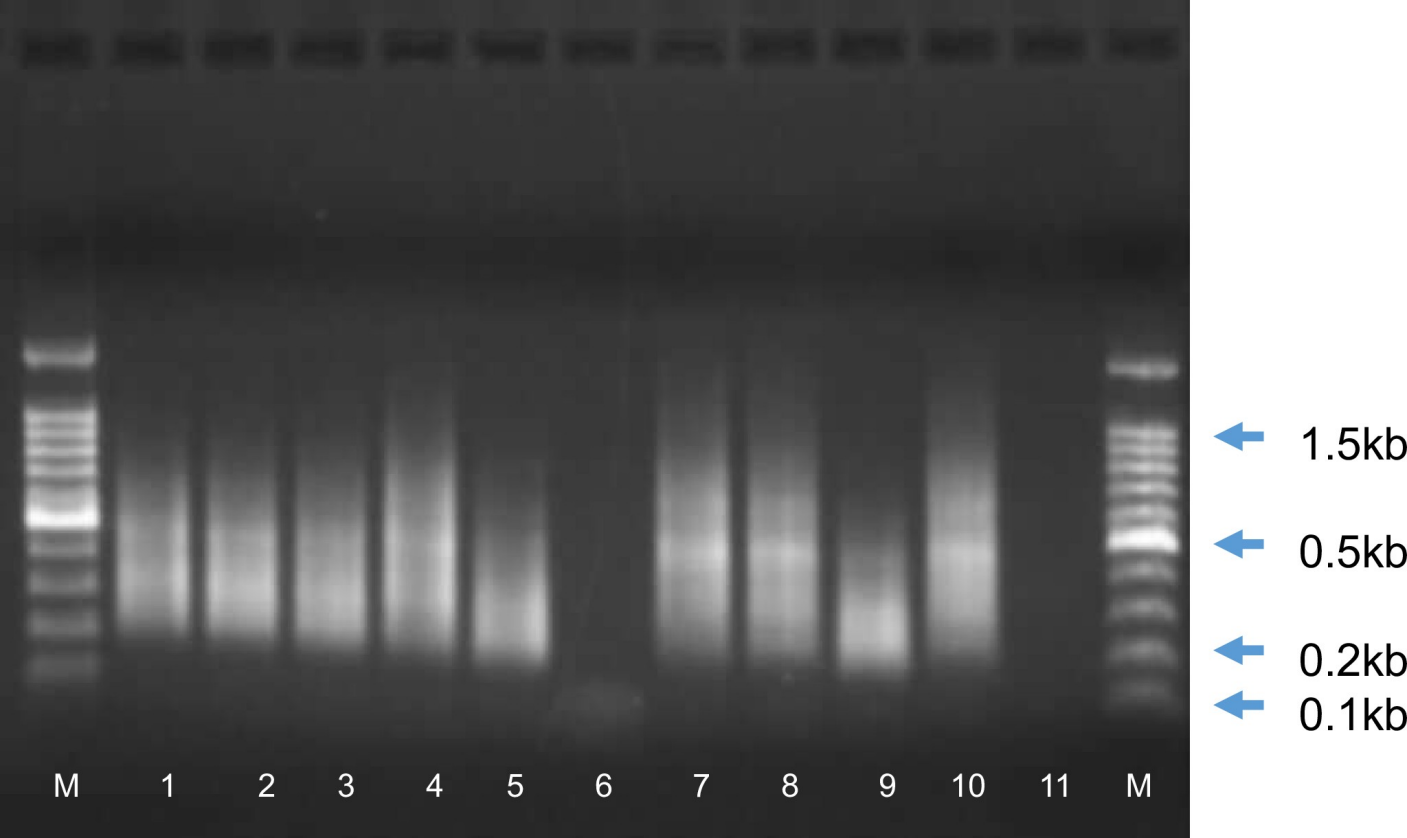
Photograph of a representative agarose gel in which SISPA products of RVF viruses were resolved. Lanes contain products as follows: lane 1: M03/10, lane 2: M15/10, lane 3: M06/10, lane 4: M19/10, lane 5: M21/10, lane 6: M22/10, lane 7: M23/10, lane 8: M25/10, lane 9: M26/10, lane 10: M33/10 lane 11: no DNA. Lanes labeled M contain DNA size markers, with corresponding sizes of some indicated in kilo basepairs (kb).

This study focused on RVF viruses isolated from animal specimens during the disease outbreaks in South Africa in the period spanning 2008 to 2010, but also included viruses from the other major outbreaks in 1955 (M1955) and 1974–1975 (M57/74 and M1975Bov). The complete genome sequences of 23 isolates were determined using a combination of SISPA [28] and Genome Sequencer 454 platform. A representative profile of SISPA products obtained from the virus isolates is shown in Fig 1. The aforementioned 23 RVFV isolates represent four of the 15 reported outbreaks in 2008, three of the 19 outbreaks reported in 2009 and six of the 484 outbreaks in 2010 (Table 1) [34, 35]. The complete genome sequence of isolate M48/08 from Madagascar was also determined (Table 1).

The nucleotide sequence data of all the RVFVs were assembled in order to generate complete S, M and L segments and were deposited in GenBank with accession numbers as indicated in Table 1.

### Coding regions under selection pressure

Sequence alignments were generated for each of the three segments using all the available RVFV sequence data in GenBank. The alignments, which included full genome sequences of 120–140 virus isolates depending on the segment, were used in evaluating the evolutionary dynamics acting on each of the three segments.

Generally, sequence diversity among the segments were <5% among S or L segments, and <6% among M segments. Bayesian coalescent estimations of RVF genomes indicated that the segments evolve at a mean rate between 3.9 x10^-4^ and 4.17 x10^-4^ substitutions per site per year, regardless of the molecular model used. This is in agreement with previous Bayesian estimations [12,20]. Similarly, the estimated Time to Most Recent Common Ancestor (TMRCA) supports previous estimations of between 1880 and 1890 [12]. In order to determine the influence of substitution rate on biological function, we estimated the effect of differential selection pressures by calculating the rate of non-synonymous (d_N_) to synonymous (d_S_) substitutions. All the coding regions were found to be under purifying selection pressure (d_N_/d_S_ <1).

### Evidence for M segment reassortment but no intragenic recombination

**Fig 2:**
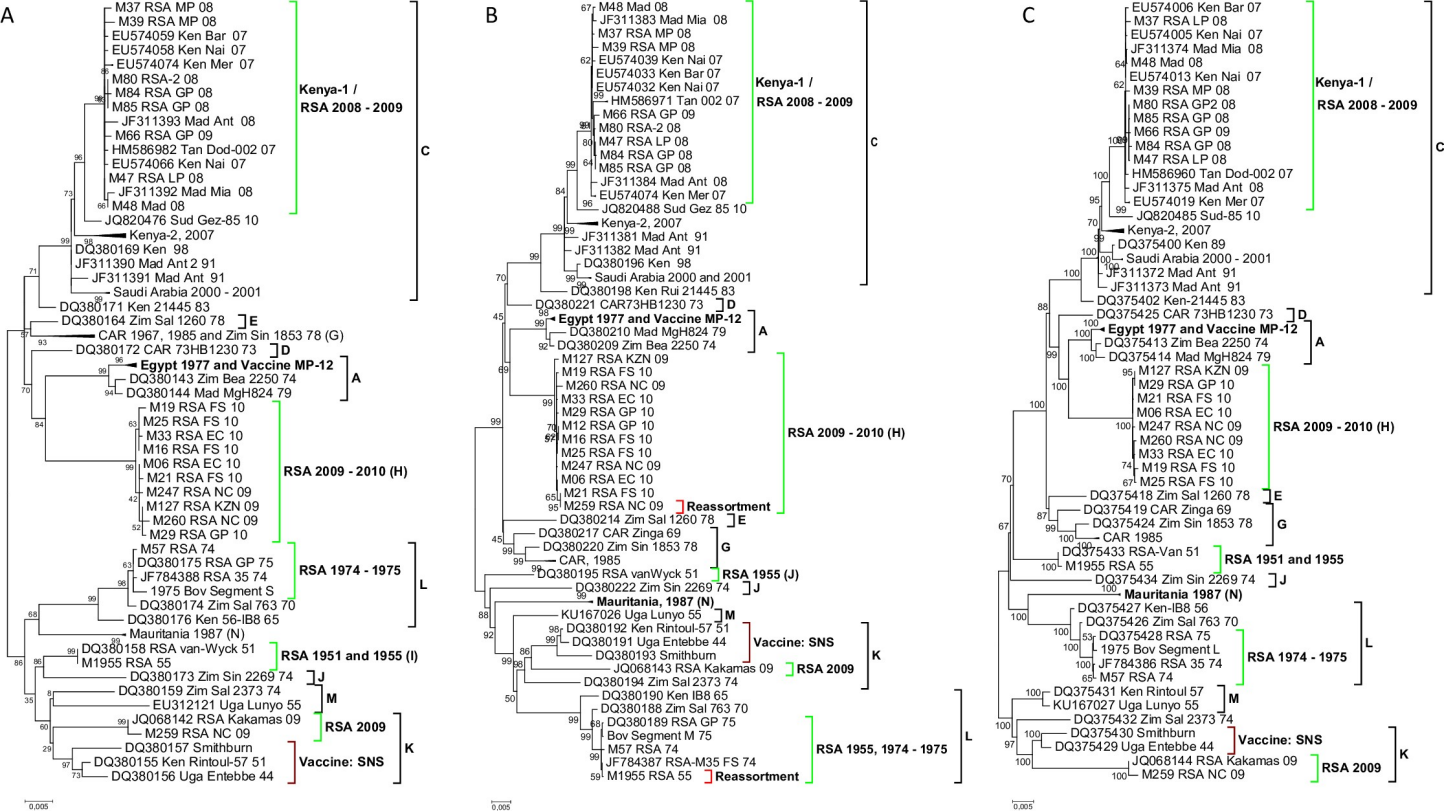
**A-C. Phylogenetic trees derived from nucleotide sequence data of the three genomic segments of RVF virus segments. Segment S (A), Segment M (B) and Segment L (C) follows the same Lineage names as described by Grobbelaar et al., 2013.** All Lineages containing RVF viruses from South Africa are presented in green. The two segment M re-assortments are indicated in red.

Using Maximum likelihood trees, the phylogenetic relationship of the 23 RVFV isolates was assessed in relation to 50 other isolates the genome sequences of which were already in GenBank (S1 Table). Incongruences among the tree topologies of the individual genome segments were observed (Fig 2A–2C), prompting an investigation of possible influence of recombination and reassortment.

We found no evidence for intragenic recombination among any of the three segments. This is probably due to the low genetic diversity among the sequences [12,30]. Reassortment has been described for RVFV [12,16] and was therefore investigated utilizing the current data.

Previous work using nucleotide sequences of the three segments assigned 15 lineages to the available RVFVs [20]. Our data indicate that one of the 2009 and all the 2008 isolates from South Africa and Madagascar (M49/08) clustered in Lineage C or Kenya-1 (Fig 2A, 2B and 2C). The remaining 2009 and 2010 isolates clustered within Lineage H [20], except isolate M259_RSA_09. The latter originated from serum of a bovine in Upington, Northern Cape Province, South Africa. Both its segments L and S cluster in Lineage K together with that of isolate JQ068143 from Kakamas (also in the Northern Cape Province); however, its segment M clustered in Lineage H along with the rest of the 2009–2010 isolates (Fig 2B). This indicates that isolate M259_RSA_09 is probably a segment reassortant from a coinfection with RVFVs in Lineages H and K. Whether this event occurred in an insect vector or an animal host is not clear.

Segments L and S of isolate M1955_RSA_55 cluster in Lineage I together with the 1951 South African Van-Wyck isolate (DQ380158) (Fig 2A and 2C). However, segment M of this isolate (M1955_RSA_55) places it in Lineage L with the isolates of the 1974 and 1975 outbreaks in South Africa (Fig 2B). Thus, isolate M1955_RSA_55 was the second RVFV in this study, which had sequence features suggesting that it may be a segment reassortant.

**Fig 3:**
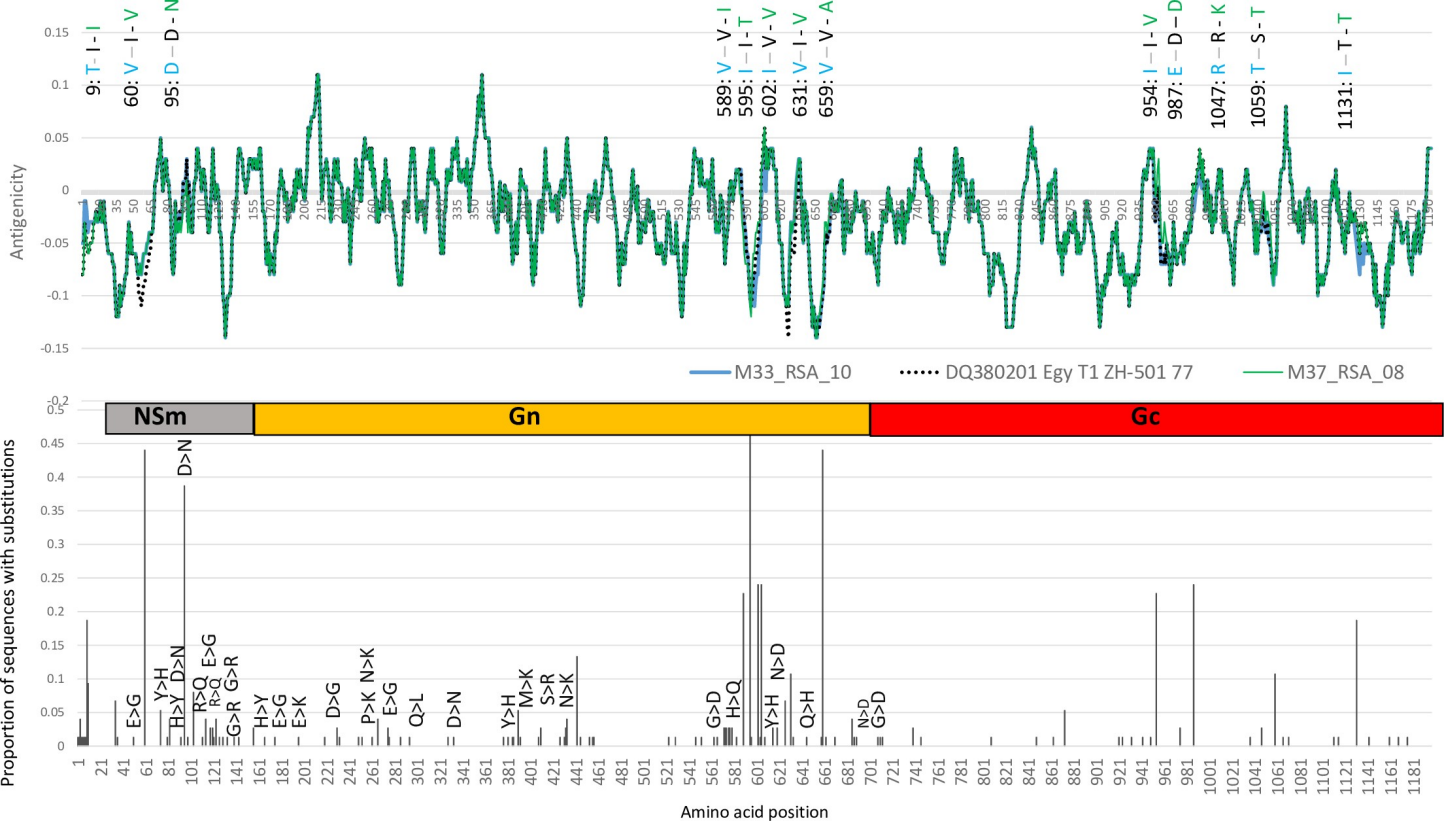
Welling antigenicity plots of proteins encoded by the M segments of isolates M33_RSA_10 in blue, ZH501-Egy-77 in black and M37_RSA_08 in green. Differences in amino acids residues among the three isolates are indicated on top of the antigenicity plots, with each isolate represented in its assigned colour. A graphical representation of the Non-structural protein (NSm), and glycoproteins (Gn) and (Gc) regions separate the antigenicity plots from the graph depicting the proportion of substitutions per amino acid position. These are representative of the 23 sequences of the M segments analyzed in this study and those of the previously published RVF viruses listed in S1 Table.

Since both these putative reassortment events relate only to Segment M, which encodes two glycoproteins (Gc and Gn), the segment was subjected to additional analysis. The amino acid sequences of the glycoproteins encoded by the M segment of different RVF virus isolates from the 2008–2010 outbreak were compared to those previously published (S1 Table). The predicted amino acid residues are conserved with <3% differences in the percentage sequence identity. Of the amino acid changes, 55% are conservative, 9.8% result in loss of a charge, 17% in gain of a charge and 2.7% in change of a charge. The positions of amino acid substitutions relative to the proportion of sequences with that change and those resulting in a change of charge are shown in Fig 3. Even though the majority of the substitutions are at the C-terminal region of the glycoprotein Gn, they are only observed in a small number of the sequences and the majority of them were conservative. One exception was a change from D (Aspartic acid) to N (Asparagine) at the amino acid position 95, which is prominent in the 2008–2009 isolates in Lineage C, Fig 2B.

To investigate the possible influence of the amino acid changes on the antigenic properties of the viruses, we performed antigenicity predictions using Welling [36], with a window size of 11. Antigenicity plots for isolate M33_RSA_10, M37_RSA_08 and T1 ZH-501 isolated in Egypt in 1977 are shown in Fig 3. These isolates represent Lineages H, C and A, respectively. Virus ZH501 from the 1977 outbreak in Egypt has been shown to be associated with increased virulence in rats [37]. Although this observation in rodents may not necessarily hold for other susceptible animal species, this isolate was included in the analysis here for comparison with isolates from Lineages H and C [20]. The major differences in antigenicity predictions between ZH501-77 and the South African isolates are at positions 60 and 631 (Fig 3). Isolate ZH501-77 has a valine at both of these positions in contrast to the South African isolates which have isoleucine. Association of virulence with amino acid substitutions at positions 595, 631, 659 and 1059 has been shown in previous studies [37].

## Discussion

South Africa has experienced three major periods of RVF outbreaks, the first in 1950–1951, the largest in 1974–1976 and lastly in 2008–2011. In 2008 a total of 15 outbreaks were reported, localized to the central provinces of Limpopo, Mpumalanga, North West and Gauteng [34]. Complete genome sequence analysis of viruses isolated from the 2008 outbreak clusters them in Lineage C together with isolates from a 2007 outbreak in Kenya, known as Lineage Kenya-1, Fig 2A, Fig 2B and Fig 2C [12,20]. In contrast to the localized outbreak of 2008, 19 outbreaks which were reported in 2009 were widespread, with single cases in Mpumalanga and Gauteng, and the rest in KwaZulu-Natal, the Eastern Cape or along the Orange River in the Northern Cape [34,38]. Similar to the isolates from the 2008 outbreaks, the 2009 isolate from Gauteng clustered in Lineage C, within Lineage Kenya-1 (Fig 2A and 2B). This 2009 Gauteng outbreak appears to have been caused by the 2008 RVF viruses present in that region. Isolates from both the geographically distinct KwaZulu-Natal and Northern Cape outbreaks of 2009, clustered in Lineage H (Fig 2A). This lineage includes isolate M259_RSA_09, a segment M reassortant, whose segments L and S cluster with the 2009 Kakamas isolate (JQ068142) in Lineage K (Fig 2B). It is therefore possible that the RVF viruses associated with the majority of the outbreaks in 2009 originated from a single source.

In 2010 a total of 484 outbreaks were reported. These were found in every province of South Africa, except KwaZulu-Natal [24]. Initial reports of outbreaks during this time were from Bultfontein and Brandfort, both in the Free State; subsequently, cases were reported from across the country [34].

Similar to the virus isolates from the 2009 outbreaks in KwaZulu-Natal and the Northern Cape, all the isolates from the 2010 outbreaks clustered in Lineage H (Fig 2A and 2B). The eight isolates from the 2010 outbreak analyzed in this study (Table 1 and Fig 2) are not necessarily statistically representatives of the 14342 cases reported in that year, but analyses of their nucleotide sequence data support speculation that the 2010 outbreak was a continuation of the 2009 in KZN and Northern Cape outbreaks. The clustering of isolates in lineage H (Fig 2A) gives an indication that new strains do evolve due to nucleotide substitution (Fig 3), albeit at slow/low rate. It is possible that these viruses were not introduced from elsewhere outside South Africa, but rather that they mutated over time and caused outbreaks when suitable conditions prevailed.

This study has contributed full genome sequence of RVFVs M57_RSA_74 isolated during the 1974 outbreaks and M1955_RSA_55 isolated from one of the 28 outbreaks in 1955 [34]. The largest RVF epidemic reported in South Africa was between 1973 and 1976, with mortality rates of 95% and cases reported from every province [34,39]. Previous studies have clustered the 1973–1975 isolates into Lineage L along with a 1970 isolate from Zimbabwe and a 1956 isolate from Kenya [12, 20]; as expected isolate M57_RSA_74 clustered with these (Fig 2A, 2B and 2C). In contrast, Segment S and Segment L of isolate M1955_RSA_55 clustered with a 1951 South African isolate known as van-Wyck in Lineage I (Fig 2A and 2C) [16], but Segment M clustered with isolates from the 1973–1976 outbreaks in Lineage L (Fig 2B), making this 1955 isolate a segment M reassortant. The occurrence of segment M reassortment in M1955_RSA_55 indicates that multiple RVF virus lineages can co-circulate, leading to reassortant viruses re-emerging decades later causing disease outbreaks.

The evolutionary dynamics of RVFVs are characterized by low substitutions rates (3.9 x10^-4^ and 4.17 x10^-4^ substitutions per site per year) under strong purifying or negative selection with the major genomic diversity resulting from reassortment [12,15,35]. Similar evolutionary dynamics have been described in other arboviruses such as bluetongue virus and Epizootic haemorrhagic disease virus, due to the obligatory replication of the virus in both its insect vector and mammalian host [40]. The majority of reasortment events described in RVFV involve the exchange of segment M, resulting in antigenic shift due to the two glycoproteins Gn and Gc encoded by this segment [12,16,20].

Although RVF virus is antigenically homogenous, some isolates of the virus differ in the severity of disease they cause in the mammalian host [37,40]. Whereas some of these differences may be attributable to the individual host, others are inherent to the virus. Differences in virulence and lethality of RVF virus isolates have been observed during the experimental infection of BHK cells [41], mice [37], sheep [42] and cattle [41]. Significant differences associated with the severity of RVF in humans have also been observed [43,44]. An increase in the severity of RVF since the 1977 outbreak in Egypt to the devastating outbreak during the 2006–2008 in East Africa have been reported [45]. Although a definitive association between genotype and lethal phenotype has not been established for RVFV, various amino acid substitutions have been implicated in this phenotype [41]. The most prominent substitution in the glycoproteins are 595 I>V, 605 R>K, 631 I>V, 659 V>A located in Gn and 1059 S>T within Gc [12]. Another variation was identified in ZH501, isolate from a human in Egypt during an outbreak in 1977, which resulted in the change of Glycine to Glutamic acid at position 277. The virus with the Glutamic acid displayed an increased virulence in mice, compared to the virus with Glycine in the same position [37].

The majority of RVF viruses analysed in this study had Glutamine at position 277, except wild type isolate 763/70 from a foetus aborted during an outbreak of the disease in Zimbabwe in 1970 [12]. This study identified additional substitutions between the lethal isolate ZH501-77 from Egypt and isolates belonging to Lineage H from 2010 in South Africa (Fig 3B). The substitutions included 602 V>I, 987 D>E and 1131 T>I. The possible influence of each of these substitutions on the pathogenicity of RVFVs remain to be investigated.

One caveat with the dataset analyzed here is that the isolates might not be representative of the RVF viruses circulating during the 2008–2010 outbreak. This is inherent in the way the study was done: samples brought for testing at the ARC-OVR are opportunistic and are not necessarily representative of cases of RVF in animals in South Africa. During this period, the RVFVs whose genomes could be analyzed were the viruses that could infect BHK 21 cells growing in culture media; and finally, good quality sequence data could not be obtained from all RVFVs, which were isolated in cell culture. A different picture of RVF viruses and their potential quasispecies might emerge when genome analyses are performed on viruses obtained directly from representative proven clinical cases. This is currently not possible in our system, but determining the entire RVF viral genome sequence directly from clinical samples is being investigated.

## Supporting information

S1 Table Previously published RVF virus genome sequences used in the study. (DOCX)

## References

[1] International Committee on Taxonomy of Viruses (ICTV)". International Committee on Taxonomy of Viruses (ICTV). Retrieved 6th August 2018

[2] Daubney R, Hudson J, Garnhann P. Enzootic hepatitis or Rift Valley fever. An undescribed virus disease of sheep, cattle and man from East Africa. J Pathol Bacteriol 1931; 34: 545–579.

[3] McIntosh BM, Russell D, dos Santos I, Gear JH. Rift Valley fever in humans in South Africa. S. Afr Med J. 1980; 58:803–806. 7192434

[4] Linthicum KJ, Davies FG, Kairo A, Bailey CL. Rift Valley fever virus (family Bunyaviridae, genus Phlebovirus). Isolations from Diptera collected during an inter-epizootic period in Kenya. J Hyg (Lond). 1985; 95:197–209. 2862206 10.1017/s0022172400062434 PMC2129511

[5] Turell MJ, Presley SM, Gad AM, Cope SE, Dohm DJ, Morrill JC, et al Vector competence of Egyptian mosquitoes for Rift Valley fever virus. Am J Trop Med Hyg. 1996; 54:136–9. 8619436 10.4269/ajtmh.1996.54.136

[6] Alexander RA. Discussion on both Rift Valley Fever (R.V.F.) and Wesselsbron Virus Disease (W.V.D.). In: Proceedings of the IVth Annual Meeting of the Inter African Advisory Committee on Epizootic Disease. 1957 p. 29 Dakar: Interafrican Bureau for Epizootic Diseases.

[7] Anyamba A, Chretien J-P, Small J, Tucker CJ, Formenty PB, Richardson JH, et al Prediction of a Rift Valley fever outbreak. Proc Natl Acad Sci U S A. 2009; 106:955–9. 10.1073/pnas.0806490106 19144928 PMC2626607

[8] Schoemaker T, Boulianne C, Vincent MJ, Pezzanite L, AI-Qahtani MM, AI-Mazrou Y, et al Genetic Analysis of viruses associated with emergence of Rift Valley fever in Saudi Arabia and Yemen, 2000–01. Emerg Infec Dis. 2002; 8: 1415–20. 12498657 10.3201/eid0812.020195 PMC2738516

[9] Morvan J, Lesbordes JL, Rollin PE, Mouden JC, Roux J. First fatal human case of Rift Valley fever in Madagascar. Trans R Soc Trop Med Hyg. 1992; 86:320 1412665 10.1016/0035-9203(92)90329-b

[10] Hassan OA, Ahlm C, Sang R, Evander M. The 2007 Rift Valley fever outbreak in Sudan. PLoS Negl Trop Dis. 2011; 5(9):e1229 10.1371/journal.pntd.0001229 21980543 PMC3181235

[11] Muller R, Saluzzo JF, Lopez N, Dreier T, Turell M, Smith J, et al Characterization of clone 13, a naturally attenuated avirulent isolate of Rift Valley fever virus, which is altered in the small segment. Am J Trop Med Hyg. 1995; 53(4):405–11. 7485695 10.4269/ajtmh.1995.53.405

[12] Bird BH, Khristova ML, Rollin PE, Ksiazek TG, Nichol ST. Complete genome analysis of 33 ecologically and biologically diverse Rift Valley fever virus strains reveals widespread virus movement and low genetic diversity due to recent common ancestry. J Virol. 2007; 81:2805–16. 10.1128/JVI.02095-06 17192303 PMC1865992

[13] Sall AA, Zanotto PM De A, Sene OK, Zeller HG, Digoutt JP, Thiongane Y, et al Genetic reassortment of Rift Valley fever virus in nature. J Virol. 1997; 3:8196–8200. 10.1128/jvi.73.10.8196-8200.1999 PMC112837 10482570

[14] Liu L, Celma CCP, Roy P. Rift Valley fever virus structural proteins: expression, characterization and assembly of recombinant proteins. Virol J. 2008; 5:82 10.1186/1743-422X-5-82 18638365 PMC2488336

[15] Bouloy M, Weber F. Molecular biology of Rift Valley fever virus. Open Virol J.2010; 4:8–14. 10.2174/1874357901004020008 20517489 PMC2878978

[16] Freire CCM, Iamarino A, Ly Soumare PO, Faye O, Sall AA, Zanotto PMA. Reassortment and distinct evolutionary dynamics of Rift Valley fever virus genomic segments. Sci Rep. 2015; 5:11353 10.1038/srep11353 26100494 PMC4477411

[17] MacOwan KD. The development of a livestock industry in Kenya. Vet Hist. 1994; 8:29–37. 11619287

[18] Hanotte O, Bradley DG, Ochieng JW, Verjee Y, Hill EW, Rege JE. African pastoralism: genetic imprints of origins and migrations. Science. 2002; 296:336–339. 10.1126/science.1069878 11951043

[19] Davies FG. Observations on the epidemiology of Rift Valley fever in Kenya. J. Hygiene 1975; 75:219–30. 1058243 10.1017/s0022172400047252 PMC2130298

[20] Grobbelaar AA, Weyer J, Leman PA, Kemp A, Paweska JT, Swanepoel R. Molecular epidemiology of Rift Valley fever virus. Emerg Infect Dis. 2011; 17:2270–6. 10.3201/eid1712.111035 22172568 PMC3311189

[21] Kegakilwe, P. S. An atypical outbreak of Rift Valley fever in the Northern Cape in October 2009. In: Proceedings of the 9th annual congress of the Southern African Society for Veterinary Epidemiology and Preventive Medicine. 18–20 August 2010. Farm Inn, Republic of South Africa

[22] Alexander RA. Rift Velley fever in the Union. J S Afr Vet Med Assoc. 1951; 22:105.

[23] Schulz KH. Rift valley fever in South Africa, Special Report No 5/51 Union Department of Health, Plague Research Laboratory, Johannesburg, South Africa, 5 1951.

[24] Pienaar NJ. A retrospective analysis of the epidemiology of Rift Valley fever in South Africa [dissertation]. Pretoria (South Africa): University of Pretoria; 2011.

[25] OIE Manual of diagnostic test and vaccines for terrestrial animals (mammals, birds and bees). Chapter 2.1.14. Rift Valley Fever. May 2008.

[26] Drosten C, Göttig S, Schilling S, Asper M, Panning M, Schmitz H, et al Rapid detection and quantification of RNA of Ebola and Marburg viruses, Lassa virus, Crimean-Congo hemorrhagic fever virus, Rift Valley fever virus, dengue virus, and yellow fever virus by real-time reverse transcription-PCR. J Clin Microbiol. 2002; 40:2323–30. 10.1128/JCM.40.7.2323-2330.2002 12089242 PMC120575

[27] von Teichman B, Engelbrecht A, Zulu G, Dungu B, Pardini A, Bouloy M. Safety and efficacy of Rift Valley fever Smithburn and Clone 13 vaccines in calves. Vaccine. 2011; 29:5771–7. 10.1016/j.vaccine.2011.05.055 21664400

[28] Djikeng A, Halpin R, Kuzmickas R, Depasse J, Feldblyum J, Sengamalay N, et al Viral genome sequencing by random priming methods. BMC Genomics. 2008; 9:5 10.1186/1471-2164-9-5 18179705 PMC2254600

[29] Thompson JD, Higgins DG, Gibson TJ. CLUSTAL W: improving the sensitivity of progressive multiple sequence alignment through sequence weighting, position-specific gap penalties and weight matrix choice. Nucleic Acids Res. 1994; 22:4673–80. 7984417 10.1093/nar/22.22.4673 PMC308517

[30] Tamura K, Peterson D, Peterson N, Stecher G, Nei M, and Kumar S. MEGA5: Molecular Evolutionary Genetics Analysis using Maximum Likelihood, Evolutionary Distance, and Maximum Parsimony Methods. Mol Biol Evol. 2011; 28: 2731–2739. 10.1093/molbev/msr121 21546353 PMC3203626

[31] Hall BG. Building phylogenetic trees from molecular data with MEGA. Mol Biol Evol. 2013; 30:1229–35. 10.1093/molbev/mst012 23486614

[32] Heath L, van der Walt E, Varsani A, Martin DP. Recombination patterns in aphthoviruses mirror those found in other picornaviruses. J Virol. 2006; 80:11827–32. 10.1128/JVI.01100-06 16971423 PMC1642601

[33] Drummond AJ, Suchard MA, Xie D, Rambaut A. Bayesian phylogenetics with BEAUTi and the BEAST 1.7. Mol Bio Evol. 2012; 29:1969–73. 22367748 10.1093/molbev/mss075 PMC3408070

[34] Pienaar NJ, Thompson PN. Temporal and spatial history of Rift Valley Fever in South Africa: 1950 to 2011. Onderstepoort J Vet Res. 2013; 80:384 10.4102/ojvr.v80i1.384 23718815

[35] Pepin M, Bouloy M, Bird BH, Kemp A, Paweska J. Rift Valley fever virus (Bunyaviridae: Phlebovirus): an update on pathogenesis, molecular epidemiology, vectors, diagnostics and prevention. Vet Res. 2010; 41:61 10.1051/vetres/2010033 21188836 PMC2896810

[36] Welling GW, Weijer WJ, van der Zee R and Welling-Wester S. Prediction of sequential antigenic regions in proteins. FEBS Letters, 1985; 188(2): 215–218. 2411595 10.1016/0014-5793(85)80374-4

[37] Morrill JC, Ikegami T, Yoshikawa-Iwata N, Lokugamage N, Won S, Terasaki K, et al Rapid accumulation of virulent Rift Valley fever virus in mice from an attenuated virus carrying a single nucleotide substitution in the mRNA. PLoS One. 2010; 5: e9986 Erratum in: PLoS One. 2010; 5(4). 10.1371/journal.pone.0009986 20376320 PMC2848673

[38] Williams R, Malherbe J, Weepener H, Majiwa P, Swanepoel R. Anomalous High Rainfall and Soil Saturation as Combined Risk Indicator of Rift Valley Fever Outbreaks, South Africa, 2008–2011. Emerg Infect Dis. 2016; 22:2054–62. 10.3201/eid2212.151352 27403563 PMC5189125

[39] Swanepoel R, Coetzer JAW. Rift Valley fever In: Coetzer JAW, Tutsin RC, editors. Infectious disease of livestock with special reference to Southern Africa. Cape Town: Oxford University Press: 2004 1037–70.

[40] Wilson WC, Gaudreault NN, Jasperson DC, Johnson DJ, Ostlund EN, Chase CL, et al Molecular evolution of American field strains of Bluetongue and Epizootic haemorrhagic disease viruses. Vet Ital. 2015; 51(4). 269–273. 10.12834/VetIt.555.2627.1 26741243

[41] Wilson WC, Davis AS, Gaudreault NN, Faburay B, Trujillo JD, Shivanna V, et al Experimental Infection of Calves by Two Genetically-Distinct Strains of Rift Valley Fever Virus. Viruses. 2016; 8(5). pii: E145. 10.3390/v8050145 27223298 PMC4885100

[42] Moutailler S, Roche B, Thiberge JM, Caro V, Rougeon F, Failloux AB. Host alternation is necessary to maintain the genome stability of Rift Valley fever virus. PLoS Negl Trop Dis. 2011; 5: e1156 10.1371/journal.pntd.0001156 21629727 PMC3101185

[43] Faburay B, Gaudreault NN, Liu Q, Davis AS, Shivanna V, Sunwoo SY, et al Development of a sheep challenge model for Rift Valley fever. Virol. 2016; 489:128–40. 10.1016/j.virol.2015.12.003 26748334

[44] Al-Hazmi M, Ayoola EA, Abdurahman M, Banzal S, Ashraf J, El-Bushra A, et al Epidemic Rift Valley fever in Saudi Arabia: a clinical study of severe illness in humans. Clin Infect Dis. 2003; 36:245–52. 10.1086/345671 12539063

[45] Baba M, Masiga DK, Sang R, Villinger J. Has Rift Valley fever virus evolved with increasing severity in human populations in East Africa? Emerg Microbes Infect. 2016; 5: e58 10.1038/emi.2016.57 27329846 PMC4932650

