# Supplementary material for: A comparative genome analysis of Rift Valley Fever virus isolates from foci of the disease outbreak in South Africa in 2008-2010
Source: PLoS Negl Trop Dis. 2019 Mar 21;13(3):e0006576. doi: 10.1371/journal.pntd.0006576 (PMC6445458; doi:10.1371/journal.pntd.0006576)
Supplement: S1 Table — (DOCX) [file pntd.0006576.s001.docx]

| Isolate Name | Country | Year | Segment L | Segment M | Segment S |
| --- | --- | --- | --- | --- | --- |
| T1: mosquito which fed on hamster infected with ZH-501 | Egypt | 1977 | DQ375407 Egy Sha T1 77 | DQ380201 Egy T1 ZH-501 77 | DQ380150 Egy T1 ZH-501 77 |
| ZH-1776 | Egypt | 1978 | DQ375411 Egy Gha ZH-1776 78 | DQ380203 Egy Gha ZH-1776 78 | DQ380153 Egy Gha ZH-1776 78 |
| ZM-657 | Egypt | 1978 | DQ375409 Egy Sha ZM-657 78 | DQ380204 Egy Sha ZM-657 78 | DQ380146 Egy Sha ZM-657 78 |
| ZS-6365 | Egypt | 1979 | DQ375410 Egy Cha ZS-6365 79 | DQ380205 Egy Gha ZS-6365 79 | DQ380145 Egy Gha ZS-6365 79 |
| ZH-548 | Egypt | 1977 | DQ375403 Egy Sha ZH-548 77 | DQ380206 Egy Sha ZH-548 77 | DQ380151 Egy Sha ZH-548 77 |
| ZC-3349 | Egypt | 1978 | DQ375412 Egy Asy ZC-3349 78 | DQ380207 Egy Asy ZC-3349 78 | DQ380152 Egy Asy ZC-3349 78 |
| 763/70 | Zimbabwe | 1970 | DQ375426 Zim Sal 763 70 | DQ380188 Zim Sal 763 70 | DQ380174 Zim Sal 763 70 |
| 2373/74 | Zimbabwe | 1974 | DQ375432 Zim Sal 2373 74 | DQ380194 Zim Sal 2373 74 | DQ380159 Zim Sal 2373 74 |
| 2250/74 | Zimbabwe | 1974 | DQ375413 Zim Bea 2250 74 | DQ380209 Zim Bea 2250 74 | DQ380143 Zim Bea 2250 74 |
| 1260/78 | Zimbabwe | 1978 | DQ375418 Zim Sal 1260 78 | DQ380214 Zim Sal 1260 78 | DQ380164 Zim Sal 1260 78 |
| 1853/78 | Zimbabwe | 1978 | DQ375424 Zim Sin 1853 78 | DQ380220 Zim Sin 1853 78 | DQ380168 Zim Sin 1853 78 |
| 2269/74 | Zimbabwe | 1974 | DQ375434 Zim Sin 2269 74 | DQ380222 Zim Sin 2269 74 | DQ380173 Zim Sin 2269 74 |
| MgH824 | Madagascar | 1979 | DQ375414 Mad MgH824 79 | DQ380210 Mad MgH824 79 | DQ380144 Mad MgH824 79 |
| 200803166 | Madagascar | 1991 | JF311372 Mad Ant 200803166 91 | JF311381 Mad Ant 200803166 91 | JF311390 Mad Ant 200803166 91 |
| 200803167 | Madagascar | 1991 | JF311373 Mad Ant 200803167 91 | JF311382 Mad Ant 200803167 91 | JF311391 Mad Ant 200803167 91 |
| 200803168 | Madagascar | 2008 | JF311374 Mad Mia 200803168 08 | JF311383 Mad Mia 200803168 08 | JF311392 Mad Mia 200803168 08 |
| 200803169 | Madagascar | 2008 | JF311375 Mad Ant 200803169 08 | JF311384 Mad Ant 200803169 08 | JF311393 Mad Ant 200803169 08 |
| OS-9 | Mauritania | 1987 | DQ375397 Mau OS-9 87 | DQ380183 Mau OS-9 87 | DQ380179 Mau OS-9 87 |
| OS-8 | Mauritania | 1987 | DQ375395 Mau OS-8 87 | DQ380185 Mau OS-8 87 | DQ380177 Mau OS-8 87 |
| OS-1 | Mauritania | 1987 | DQ375398 Mau OS-1 87 | DQ380186 Mau OS-1 87 | DQ380180 Mau OS-1 87 |
| Hv-B375 | Central African Republic | 1985 | DQ375422 CAR Mba Hv-B375 85 | DQ380218 CAR Aba Hv-B375 85 | DQ380161 CAR Hv-B375 85 |
| CAR-R1622 | Central African Republic | 1985 | DQ375423 CAR Ban R1622 85 | DQ380219 CAR Ban R1622 85 | DQ380160 CAR R1622 85 |
| 73HB1230 | Central African Republic | 1973 | DQ375425 CAR 73HB1230 73 | DQ380221 CAR 73HB1230 73 | DQ380172 CAR 73HB1230 73 |
| Zinga | Central African Republic | 1969 | DQ375419 CAR Zinga 69 | DQ380217 CAR Zinga 69 | DQ380167 CAR Zinga 69 |
| Kenya 56 (IB8) | Kenya | 1965 | DQ375427 Ken-IB8 56 | DQ380190 Ken-IB8 65 | DQ380176 Ken 56-IB8 65 |
| Kenya 57 (Rintoul) | Kenya | 1951 | DQ375431 Ken Rintoul 57 | DQ380192 Ken Rintoul-57 51 | DQ380155 Ken Rintoul-57 51 |
| Kenya 9800523 | Kenya | 1998 | DQ375400 Ken 9800523 98 | DQ380196 Ken 9800523 98 | DQ380169 Ken 9800523 98 |
| Kenya 83 (21445) | Kenya | 1983 | DQ375402 Ken Rui 21445 83 | DQ380198 Ken Rui 21445 83 | DQ380171 Ken Rui 21445 83 |
| 2007004194 | Kenya | 2007 | EU574004 Ken Kia 2007004194 07 | EU574031 Ken Kia 2007004194 07 | EU574057 Ken Kia 2007004194 07 |
| 2007004193 | Kenya | 2007 | EU574005 Ken Nai 2007004193 07 | EU574032 Ken Nai 2007004193 07 | EU574058 Ken Nai 2007004193 07 |
| 2007003644 | Kenya | 2007 | EU574006 Ken Bar 2007003644 07 | EU574033 Ken Bar 2007003644 07 | EU574059 Ken Bar 2007003644 07 |
| 2007002060 | Kenya | 2007 | EU574013 Ken Nai 2007002060 07 | EU574039 Ken Nai 2007002060 07 | EU574066 Ken Nai 2007002060 07 |
| 2007001564 | Kenya | 2007 | EU574017 Ken Mur 2007001564 07 | EU574044 Ken Mur 2007001564 07 | EU574072 Ken Mur 2007001564 07 |
| 2007001292 | Kenya | 2007 | EU574019 Ken Mer 2007001292 07 | EU574046 Ken Meru 2007001292 07 | EU574074 Ken Mer 2007001292 07 |
| Saudi 2000-10911 | Saudi Arabia | 2000 | DQ375401 SA 10911 00 | DQ380197 Saudi 10911 00 | DQ380170 Saudi 10911 00 |
| SA01-1322 | Saudi Arabia | 2001 | KX096941 SA 1322 01 | KX096942 Saudi 1322 01 | KX096943 Saudi 1322 01 |
| SA-75 | South Africa | 1975 | DQ375428 RSA 75 | DQ380189 RSA 75 | DQ380175 RSA 75 |
| SA-51 (Van Wyck) | South Africa | 1951 | DQ375433 RSA VanWyck 51 | DQ380195 RSA VanWyck 51 | DQ380158 RSA VanWyck 51 |
| M35/74 | South Africa | 1974 | JF784386 RSA M35 74 | JF784387 RSA M35 74 | JF784388 RSA M35 74 |
| Kakamas | South Africa | 2009 | JQ068144 RSA Kakamas 09 | JQ068143 RSA Kakamas 09 | JQ068142 RSA Kakamas 09 |
| Sudan 85-2010 | Sudan | 2010 | JQ820485 Sud Gez-85 10 | JQ820488 Sud Gez-85 10 | JQ820476 Sud Gez-85 10 |
| Sudan 86-2010 | Sudan | 2010 | JQ820484 Sud Gez 86 10 | JQ820489 Sud Gez 86 10 | JQ820477 Sud Gez 86 10 |
| Sudan 2V-2007 | Sudan | 2007 | JQ820483 Sud WNS-2V 07 | JQ820490 Sud WNS 2V 07 | JQ820472 Sud WNS-2V 07 |
| Sudan 28-2010 | Sudan | 2010 | JQ820486 Sud Gez-28 10 | JQ820491 Sud Gez 28 10 | JQ820474 Sud Gez 28 10 |
| TAN/Tan-001/07 | Tanzania | 2007 | HM586959 TAN Tan-001 07 | HM586970 Tan 001 07 | HM586981 Tan Tan-001 07 |
| TAN/Dod-002/07 | Tanzania | 2007 | HM586960 Tan Dod-002 07 | HM586971 Tan 002 07 | HM586982 Tan Dod-002 07 |
| Tan 2007000323 | Tanzania | 2007 | JF326189 Tan 2007000323 07 | JF326194 Tan 2007000323 07 | JF326203 Tan 2007000323 07 |
| Entebbe | Uganda | 1944 | DQ375429 Uga Entebbe 44 | DQ380191 Uga Entebbe 44 | DQ380156 Uga Entebbe 44 |
| Smithburn | Uganda | 1944 | DQ375430 Smithburn | DQ380193 Smithburn | DQ380157 Smithburn |
| Lunyo | Uganda | 1955 | KU167027 Uga Lunyo 55 | KU167026 Uga Lunyo 55 | EU312121 Uga Lunyo 55 |
